# The Effect of Ingesting Carbohydrate and Proteins on Athletic Performance: A Systematic Review and Meta-Analysis of Randomized Controlled Trials

**DOI:** 10.3390/nu12051483

**Published:** 2020-05-20

**Authors:** Lotte Lina Kloby Nielsen, Max Norman Tandrup Lambert, Per Bendix Jeppesen

**Affiliations:** 1 Department of Public Health, Section of Sport Science, Aarhus University, Dalgas Avenue 4, 8000 Aarhus, Denmark; lotte.lina.nielsen@gmail.com; 2 Department of Clinical Medicine, Aarhus University Hospital, Palle Juul-Jensens Boulevard 165, 8200 Aarhus N, Denmark; mntl@clin.au.dk

**Keywords:** sports nutrition, athletic performance, protein, carbohydrate, time-to-exhaustion, time-trial

## Abstract

Endurance athletes participating in sporting events may be required to complete multiple training sessions a day or on successive days with a limited recovery time. Nutritional interventions that enhance the restoration of endogenous fuel stores (e.g., liver and muscle glycogen) and improve muscle damage repair have received a lot of attention. The purpose of this review is to investigate the effect of ingesting carbohydrate (CHO) and protein (PRO) on athletic performance. Studies were identified by searching the electronic databases PubMed and EMBASE. Random-effects meta-analyses were conducted to examine the intervention efficacy. A total of 30 randomized controlled trials (RCT), comprising 43 trials and 326 participants in total, were included in this review. The meta-analysis showed an overall significant effect in Time-To-Exhaustion (TTE) and Time-Trial (TT) performance, when ingesting carbohydrates and proteins (CHO-PRO) compared to CHO-only (*p* = 0.03 and *p* = 0.0007, respectively). A subgroup analysis demonstrated a significant effect in TTE by ingesting CHO-PRO compared to CHO, when supplements were provided during and/or following an exercise bout. CHO-PRO significantly improved TTE compared to CHO-only, when a long-term recovery (i.e., ≥8 h) was implemented (*p* = 0.001). However, no effect was found when the recovery time was short-term (i.e., ≤8 h). No significant effect was observed when CHO-PRO and CHO-only supplements were isocaloric. However, a significant improved TTE was evident with CHO-PRO compared to CHO-only, when the supplements were matched for carbohydrate content (*p* < 0.00001). In conclusion, co-ingesting carbohydrates and proteins appears to enhance TTE and TT performance compared to CHO-only and presents a compelling alternate feeding strategy for athletes.

## 1. Introduction

Endogenous carbohydrate (CHO) is stored as liver and muscle glycogen [1,2,3]. Glycogen is a branched polymer of glucose where cells store and utilize glucose to meet their energetic demands. Muscle glycogen is a major energy source during prolonged moderate-to-high intensity exercise [4,5]. The development of fatigue during exhaustive exercise is often associated with low muscle glycogen concentrations [6,7]. Moreover, recent studies [6,7] have reported a relationship between low glycogen content and reduced calcium (Ca^2+^) release from sarcoplasmic reticulum (SR), an association ultimately leading to fatigue. Muscle glycogen levels are usually restored to pre-exercise levels within 24 h, if a sufficient amount of carbohydrate (CHO) is provided [1,2]. The restoration of glycogen requires the translocation of glucose transporter carrier protein-4 isoform (GLUT-4) from the intracellular domain to the plasma membrane. GLUT-4 isoform is expressed in skeletal muscle and its translocation is stimulated directly by muscle contraction and/or by circulating plasma insulin binding to its receptor [2].

Nutritional interventions that enhance the restoration of endogenous fuel stores (e.g., liver and muscle glycogen) and improve muscle damage repair have received a lot of attention [1,2,3]. Nutritional recommendations for endurance athletes advocate a high carbohydrate availability for post-exercise recovery to replenish the glycogen stores [3]. A CHO intake of 1.2–1.5 g·kg^−1^·hr^−1^ during short-term recovery (i.e., ≤8 h), appears to be the threshold in maximizing post-exercise rate of glycogen synthesis [2,3]. Moreover, it has been suggested that an immediate (i.e., within ~30 min post-exercise) consumption of CHO at frequent intervals (i.e., with ~15–30 min) may maximize muscle glycogen synthesis by maintaining high levels of plasma glucose and insulin [2,8]. In line with this, Ivy et al. [9] demonstrated lower glycogen levels, when CHO supplementation was delayed with 2 h after cessation of exercise when compared to an immediate intake of CHO.

Moreover, for athletes participating in endurance events lasting one hour or more, an intake during performance is recommended to maintain a high muscle glycogen synthesis [3]. Nutritional recommendation suggests a consumption of CHO of approximately 30–60 g/h at 15–20 min intervals throughout the first two hours of exercise [3]. These guidelines are especially important for longer endurance events, when athletes have not carbohydrate-loaded, consumed pre-exercise meals, or restricted energy intake for weight loss [3]. The primary aim of nutrient supplementation during exercise is to replace fluid loss and maintain high levels of plasma glucose [3]. A wealth of research has examined the effect of carbohydrate intake during and/or post-exercise [10,11,12,13,14,15,16,17,18,19], most of which show an improved performance.

To date, several methodological differences exist across studies investigating the effect of co-ingesting CHO and proteins, including number of calories provided, depletion protocol, and recovery duration. In some studies, the treatments were matched for carbohydrate content [15,16], while others matched the supplementations for caloric content [10,11,17]. Some studies [15,18] did not include a preceding exercise bout to deplete muscle glycogen levels, while others [10,11,13,14,16,17] did include a depletion ride. Furthermore, the majority of trials have investigated the effect of carbohydrates and proteins (CHO-PRO) when recovery time is limited [11,13,14,19,20]. Under these circumstances it may not be possible to restore substrate losses and promote muscle damage repair [2]. Whereas only a few trials [10,16,17] have examined the effect of CHO-PRO, when recovery time ranged between 12–18 h. These methodological differences across investigations could explain the inconsistent performance outcome reported.

The primary purpose of the present systematic review and meta-analysis was therefore to investigate the effect of co-ingestion of CHO-PRO on athletic performance compared to a carbohydrate-only control product. The paper further investigates the effects of CHO-PRO taken during exercise and recovery compared to CHO-only. The athletic performance was defined as cycling or running Time-to-Exhaustion (TTE). In addition, we also investigated the effect of CHO-PRO on running or cycling Time-Trial performance (TT) compared to CHO-only. Sub-analyses were performed to investigate the effect of timing of ingestion (i.e., during recovery and/or exercise) and caloric content of the supplement during TTE.

## 2. Materials and Methods

### 2.1. Literature Search

Potential research studies were identified by systematically searching the online databases PubMed (Medline) and Embase from June to October 2019. The publication dates ranged from: 01.01.1950–31.10.2019. The search terms used to identify potential research studies included: carbohydrate, protein, performance, time to exhaustion, time trial, cycling, running, athletes, and healthy (see Appendix S1 for specific search terms). Quotation marks were used to search for an exact sentence and the star symbol (*) was used to include the derivatives of a search term.

**Figure 1:**
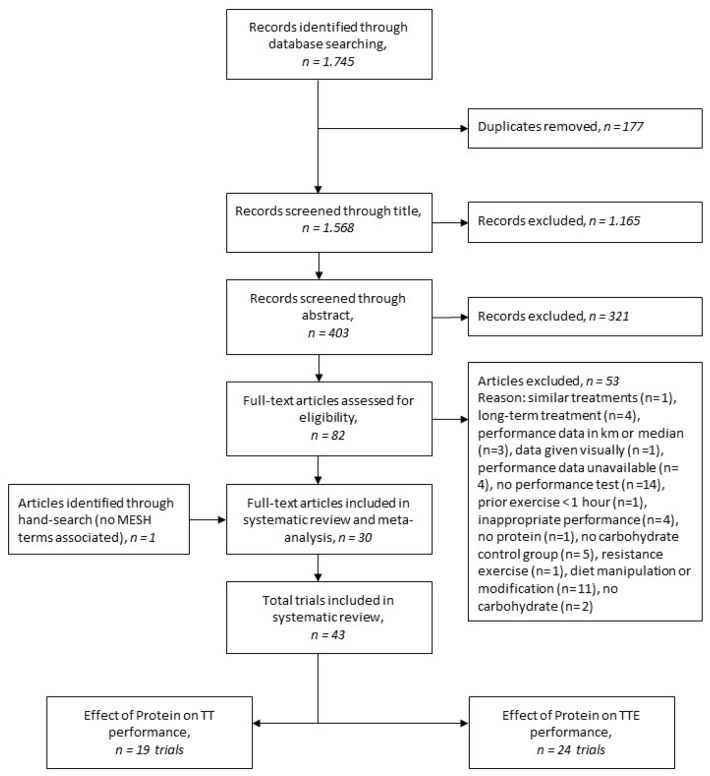
Flowchart presenting the number of studies screened, assessed for eligibility, and included in meta-analysis investigating the effect of carbohydrates and proteins (CHO-PRO) on Time-To-Exhaustion (TTE) and Time-Trial (TT) performance.

Initially, all the records were screened for relevant titles. All irrelevant titles were discarded. The remaining research studies were systematically screened for eligibility by abstract and full text, respectively. The selection process is demonstrated in the PRISMA flow chart Figure 1.

### 2.2. Inclusion and Exclusion Criteria

All studies eligible for the meta-analysis had to be designed as randomized clinical trials (RCTs). RCTs were included if they investigated the effect of co-ingestion of carbohydrates and proteins (CHO-PRO) in human subjects. Study participants had to be healthy, ≥18 years with no medical conditions, including males and/or females. The clinical trials had to include a control product consisting of CHO only. Studies providing interventions during exercise and/or during a recovery period were included in order to provide data that more accurately reflects supplement usage during endurance sporting events. A recovery period was defined as a period of time separating two exercise bouts. Both short-term (i.e., ≤8 h) and long-term (i.e., ≥8 h) recovery periods were included [1]. Studies had to assess athletic performance as TTE or TT with results expressed in time (e.g., minutes). If the TT performance was less than 10 km, the duration of a prior exercise bout to deplete the muscle glycogen had to be ≥1 h. This was to ensure that sufficient depletion of the muscle glycogen levels was likely achieved.

Articles were excluded if the research studies included participants with a medical condition (e.g., diabetics or cancer patients) or if the subjects did not match the age (e.g., children, adolescents). Studies were excluded if no athletic performance was measured, if the athletic performance was defined as, e.g., resistance exercise or if the articles were presented in other languages than English.

Studies were not excluded based on the durations of the recovery periods or on the types of CHO and proteins used in the intervention and control products.

### 2.3. Methodological Quality Assessment

**Figure 2:**
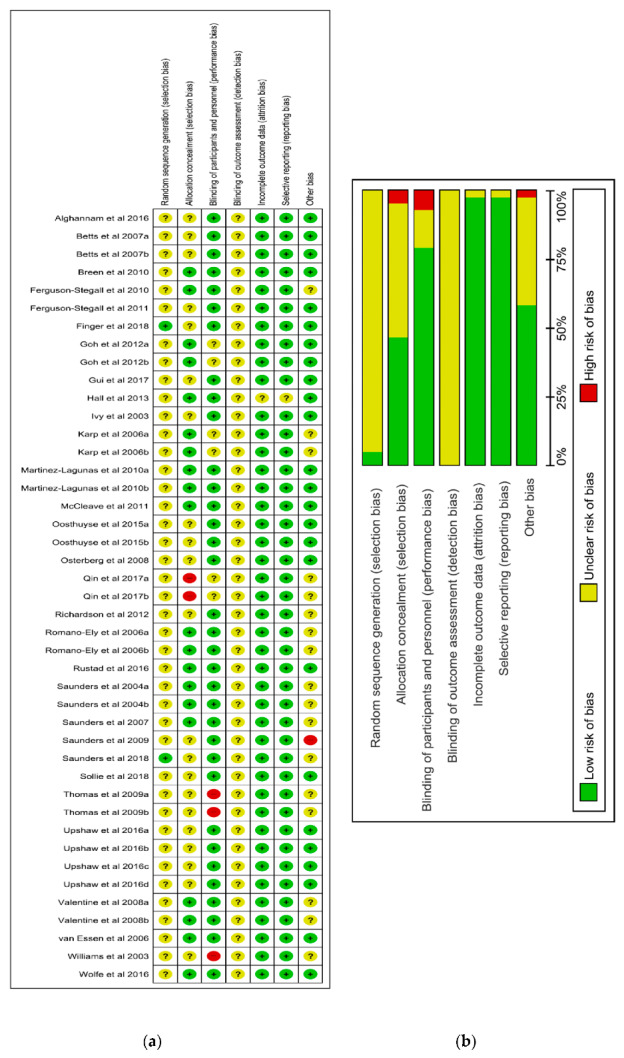
Risk of bias summary of investigations included in meta-analysis. (+): low risk; (?): unknown risk; (-): high risk. (**a**) summarising the risk assessment of each trial. (**b**) illustrating the percentage distribution of the risk assessment.

Included studies were examined for risk of bias by using the Cochrane Collaborations risk-of-bias tool. Data concerning risk of bias were extracted and included in the characteristics of the studies. This was done in Review Manager (RevMan) 5.3. The studies were evaluated for unclear, low, or high risk of bias regarding random sequence generation, allocation concealment, blinding of participants and investigators, incomplete outcome data, selective reporting, and other bias (Figure 2). A funnel plot of publication bias for all TTE and TT articles is presented in Supplemental Figures S1 and S2.

### 2.4. Data Extraction

Two reviewers (M.L. and L.N.) independently screened the research studies obtained from electronic databases to identify relevant texts. Initially all irrelevant titles were discarded. The remaining articles were systematically screened for eligibility by abstract and full-text, respectively.

Data were extracted from relevant research papers. Information extracted included: number of subjects and gender, study design, recovery duration, information on intervention and control products provided (i.e., content, amount of CHO and protein provided and timing), mode of performance (i.e., cycling or running), type of performance (i.e., TTE or TT), and result (i.e., time in minutes) (Table 1). To resolve cases of potential conflict in assessments, these were independently evaluated by PBJ.

### 2.5. Statistical Analysis

Data presented as mean ± SD of performance test for intervention and control supplements were pooled in RevMan 5.3. This was done to compare the effect of CHO-PRO on performance with CHO by inverse variance and random-effect model. In studies presenting mean ± SEM, SDs were calculated from the reported SEMs. Mean differences and 95% CI across studies were obtained by producing forest plots. Heterogeneity was assessed by using I^2^ statistics, where I^2^ values of 25%, 50%, and 75% indicated low, medium, and high heterogeneity, respectively. An I^2^ > 50% indicated significant heterogeneity between studies. 

## 3. Results

### 3.1. Overview of Included Studies

The literature search identified *n* = 1568 non duplicate studies obtained from PubMed and EMBASE (Figure 1). A total of *n* = 1165 records was excluded by title and *n* = 321 by abstract review (Figure 1). Fifty-three studies were excluded in full-text assessment, reasons included: no performance test was assessed (*n* = 14), no carbohydrate control group (*n* = 5), exercise defined as resistance exercise (*n* = 1) and/or diet manipulation or modification was implemented (*n* = 11) (Figure 1). One article was hand-searched as it did not have any EM-tree identifiers or MESH terms assigned to it.

#### 3.1.1. Subjects

A total of 30 RCT studies [10,11,12,13,14,15,16,17,18,19,20,21,22,23,24,25,26,27,28,29,30,31,32,33,34,35,36,37,38,39] comprising 43 performance test trials and 326 participants in total were included in this review (Table 1). They all included healthy participants ≥18 years. The majority of the investigations included male participants. In total approximately 79.8% of the participants were males and ~20.2% were females (Table 1).

#### 3.1.2. Study Protocol

All studies were of cross-over design, except one which was a parallel study [39]. Included investigations varied in protocol designs (e.g., duration of recovery, timing of supplementation and mode of exercise) (Table 1). Approximately 80% of the studies included a prior depletion exercise of which ~67% included a recovery period in their protocol (Table 1).

The majority of the investigations conducted exercise protocols on a bike, including cyclists and/or triathletes, whereas four studies [13,30,38,39] assessed a running performance including, e.g., recreationally active males.

#### 3.1.3. Intervention and Control Products

The combined ingestion of CHO-PRO was used as the intervention for all studies. This was compared to a control product, which contained carbohydrates only.

The amount and type of CHOs and proteins (PROs) provided during the experimental trials varied across studies. Most studies (*n* = 18 studies) [10,11,12,13,16,17,18,20,21,22,24,26,30,31,32,35,36,37] provided whey as the protein source, four studies provided chocolate milk [19,20,25,29], three gave casein [27,28,37], one trial provided milk-based (i.e., casein and whey) [29], and two trials provided plant-based protein [29], whilst six studies [14,15,23,33,38,39] did not specify which protein source was provided. The source of CHO varied across investigations. Four trials provided only dextrose [21,22,24,29], other four trials provided sucrose only [13,26,30,32] one trial gave maltodextrin [38], five studies did not describe which CHO source was administered [16,19,23,25,39] and one study gave glucose polymer [33]. The majority of trials provided a mix of different CHO sources. Five studies provided a mixture of glucose and maltodextrin [10,27,28,31,35], two gave a mix of dextrose, fructose, and maltodextrin [24,36], two provided maltodextrin and fructose [15,37], while the rest supplied CHO in the form of: sucrose and dextrose [17], glucose, fructose, and sucrose [14], sucrose and maltodextrin [18], sucrose, fructose, dextrose, and complex CHO [11], and glucose, fructose, sucrose, and trehalose [12].

Regarding the ratio of CHO:PRO provided: in six studies the CHO:PRO was not available [12,15,24,26,27,28]. The ratio of CHO:PRO also varied across trials. The majority of trials administered 4:1 of CHO:PRO [11,14,16,17,18,19,21,23,25,29,37,38,39], six gave 3:1 of CHO:PRO [13,20,32,33,34,36] and three trials gave 2:1 of CHO:PRO [10,31,35]. The rest of the trials provided in the ratios of CHO:PRO are as follows: 1.5:1 [29], 6:1 [29], 4:2 [30], 2.5:1 [22], and one trial administered 1:6.8 of CHO:PRO [35]. The following studies stated specific ratios of CHO:PRO [16,22,29,36,38], for some studies CHO:PRO ratio was estimated based on the CHO and/or PRO provided [10,11,13,14,17,18,19,20,21,23,25,30,31,32,33,34,35,37,39].

### 3.2. Effect of Carbohydrate and Protein (CHO-PRO) vs. Carbohydrate (CHO) Supplementation on Time-To-Exhaustion (TTE)

**Figure 3:**
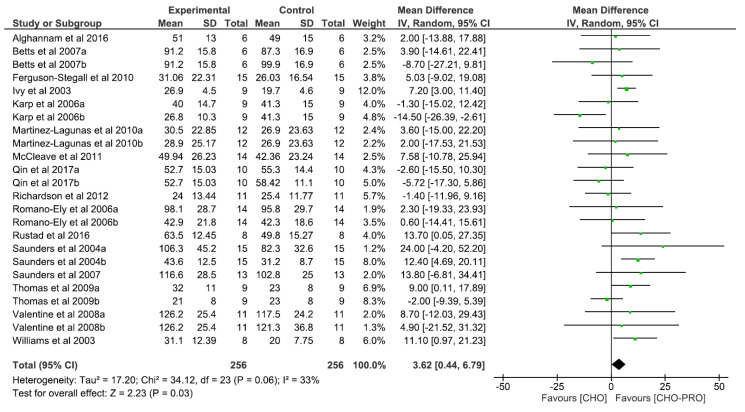
Effect of CHO-PRO on TTE performance compared to carbohydrate (CHO). CI: confidence interval; MD: mean difference.

A total of 24 trials (*n* = 256 participants) derived from 16 publications investigated the effect of CHO-PRO on time-to-exhaustion performance. A significant overall effect was found between the ingestion of CHO-PRO and CHO on TTE performance (MD: 3.62, CI: 0.44, 6.79, *p* = 0.03), favouring the intake of CHO-PRO with an overall effect size of Z = 2.23 min (Figure 3). A moderate heterogeneity was present across trials I^2^ = 33% (*p* = 0.06).

### 3.3. Effect of CHO-PRO vs. CHO Intake during TTE on Performance

Nine trials (*n* = 121 participants) examined the effect of CHO-PRO supplementation on TTE performance, when provided during exercise. This was compared to CHO.

**Figure 4:**
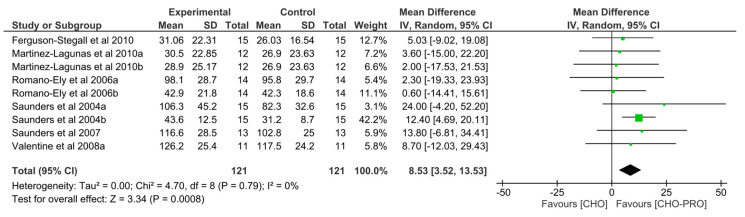
Effect of CHO-PRO intake during exercise on TTE performance compared to CHO. CI: confidence interval; MD: mean difference.

A significant overall effect was found between the ingestion of CHO-PRO and CHO on TTE performance (MD: 8.53, CI: 3.52, 13.53, *p* = 0.0008), favouring the intake of CHO-PRO with an overall effect size of Z = 3.34 min (Figure 4). A low heterogeneity was present across trials I^2^ = 0% (*p* = 0.79).

### 3.4. Effect of CHO-PRO vs. CHO Intake during Recovery on Subsequent TTE

**Figure 5:**
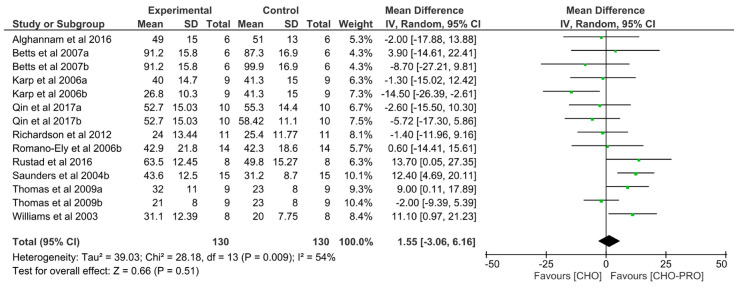
Effect of CHO-PRO intake during recovery on TTE performance compared to CHO. CI: confidence interval; MD: mean difference.

Fourteen trials (*n* = 130 participants) examined the effect of CHO-PRO supplementation on TTE performance, when provided during a recovery period. This was compared to CHO. No significant overall effect was found between the ingestion of CHO-PRO and CHO on TTE performance (MD: 1.55, CI: −3.06, 6.16, *p* = 0.51) (Figure 5). A significant heterogeneity was present across trials I^2^ = 54% (*p* = 0.009).

#### 3.4.1. Effect of CHO-PRO vs. CHO Intake during Short-Term Recovery on Subsequent TTE

11 trials (*n* = 93 participants) examined the effect of CHO-PRO supplementation on TTE performance, when provided during a short-term recovery period. This was compared to CHO.

**Figure 6:**
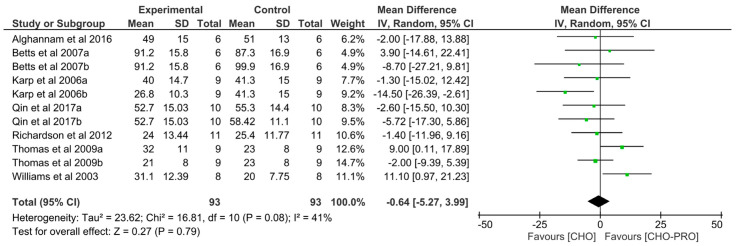
Effect of CHO-PRO intake during short-term recovery on TTE performance compared to CHO. CI: confidence interval; MD: mean difference.

No significant overall effect was found between the ingestion of CHO-PRO and CHO on TTE performance (MD: −0.64, CI: −5.27, 3.99, *p* = 0.79) (Figure 6). A moderate heterogeneity was present across trials I^2^ = 41% (*p* = 0.08).

#### 3.4.2. Effect of CHO-PRO vs. CHO Intake during Long-Term Recovery on Subsequent TTE

**Figure 7:**
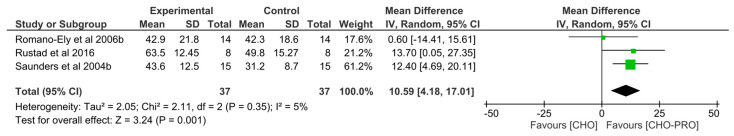
Effect of CHO-PRO intake during long-term recovery on TTE performance compared to CHO. CI: confidence interval; MD: mean difference.

Three trials (*n* = 37 participants) derived from three publications examined the effect of CHO-PRO supplementation on TTE performance, when conducting a long-term (i.e., >8 h) recovery. This was compared to CHO. A significant overall effect was found between the ingestion of CHO-PRO and CHO on TTE performance (MD: 10.59, CI: 4.18, 17.01, *p* = 0.001), favouring the ingestion of CHO-PRO during recovery periods >8 h, with an overall effect size of Z = 3.24 min (Figure 7). A low heterogeneity was present across trials I^2^ = 5% (*p* = 0.35). It should be noted, that Romano-Ely et al. 2006b [17] and Saunders et al. 2004b [16] provided supplementations both during exercise as well as during the recovery period between two exercise bouts.

### 3.5. Effect of Isocaloric Supplementation (i.e., CHO-PRO vs. CHO) on TTE Performance

**Figure 8:**
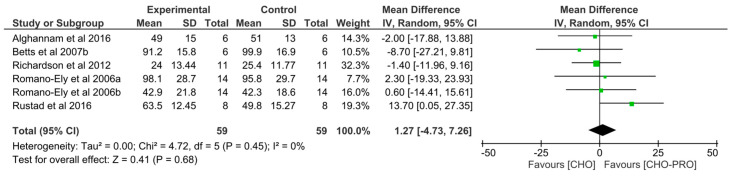
Effect of isocaloric supplementation of CHO-PRO vs. CHO on TTE performance. CI: confidence interval; MD: mean difference.

Six trials (*n* = 59 participants) derived from five publications examined the effect of isocaloric supplementation of intervention and control product on TTE performance. No significant effect was found between the ingestion of isocaloric CHO-PRO and CHO on TTE performance (MD: 1.27, CI: −4.73, 7.26, *p* = 0.68) (Figure 8). A low heterogeneity was present across trials I^2^ = 0% (*p* = 0.45).

### 3.6. Effect of Non-Isocaloric CHO-PRO vs. CHO Supplementation on TTE Performance

**Figure 9:**
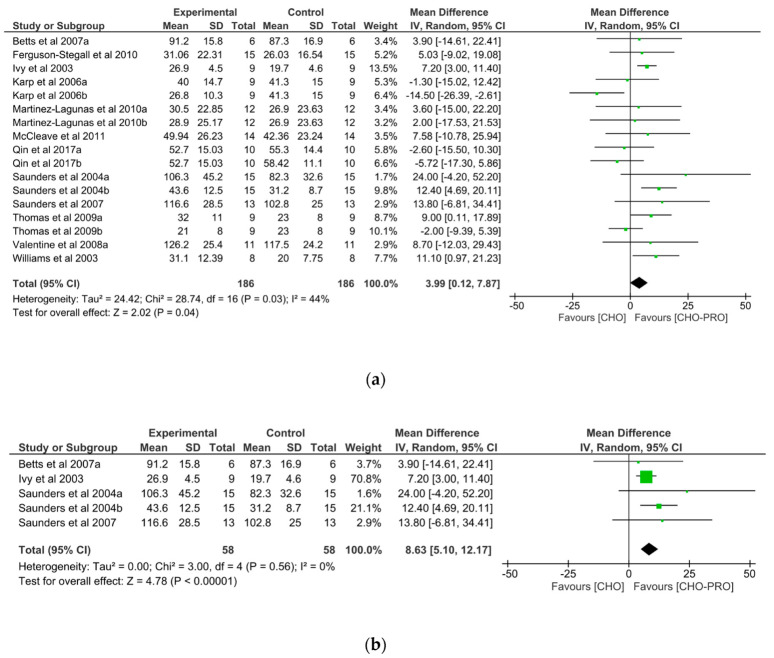
(**a**) Effect of a non-isocaloric supplementation of CHO-PRO vs. CHO on TTE performance. CI: confidence interval; MD: mean difference. (**b**) Effect of a carbohydrate-matched supplementation of CHO-PRO vs. CHO on TTE performance. CI: confidence interval; MD: mean difference.

Seventeen trials (*n* = 186 participants) derived from 12 publications examined the effect of a non-isocaloric supplementation of intervention and control product on TTE performance. A significant effect was found between the ingestion of non-isocaloric CHO-PRO and CHO on TTE performance (MD: 3.99, CI: 0.12, 7.87, *p* = 0.04), favouring the intake of CHO-PRO with an overall effect size of Z = 2.02 min (Figure 9a). A moderate heterogeneity was present across trials I^2^ = 44% (*p* = 0.03).

When doing a subgroup-analysis looking at the effect of CHO-PRO matched for volumetric content with the control product no significant effect was found (*p* > 0.5). However, when supplements were matched for CHO content (i.e., iso-CHO) a significant effect was evident favouring the ingestion of CHO-PRO compared to CHO-only on TTE performance (*p* < 0.00001) (Figure 9b).

### 3.7. Effect of CHO-PRO vs. CHO Supplementation on Time-Trial (TT) Performance

**Figure 10:**
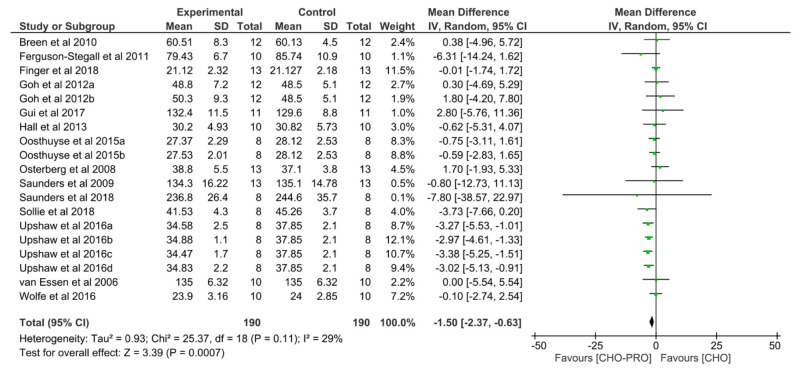
Effect of CHO-PRO on TT performance compared to CHO. CI: confidence interval; MD: mean difference.

Nineteen trials (*n* = 190 participants) derived from 14 publications examined the effect of a CHO-PRO on TT performance compared to CHO. A significant effect was found between the ingestion of CHO-PRO and CHO on TT performance (MD: −1.50, CI: −2.37, −0.63, *p* = 0.0007), favouring the intake of CHO-PRO with an overall effect size of Z = 3.39 min (Figure 10). A moderate heterogeneity was present across trials I^2^ = 29% (*p* = 0.11).

## 4. Discussion

The primary objective of the present systematic review and meta-analysis was to present the effect of co-ingesting carbohydrates and proteins on TTE performance when comparing to carbohydrate only. The key finding was that the ingestion of CHO-PRO significantly improves the overall effect in TTE performance compared to CHO-only by 2.23 min (Figure 3).

Co-ingestion of carbohydrates and proteins has gained attention as an alternate feeding strategy during limited recovery time (i.e., ≤8 h), as specific amino acids (AA) and proteins may have a potential effect in restoring the glycogen stores via insulin-mediated-pathways [10,14,40] and may promote muscle damage repair [41]. Many investigations have reported an effect with CHO-PRO on TTE performance, when comparing to CHO-only. However, not all investigations [11,12,17,20,21,22,26,28,32,33,36,38,39] report additional improvements in athletic performance with CHO-PRO compared to CHO. Differences in protocol design across studies likely explain inconsistencies in findings of CHO-PRO supplementation. Differences in the duration of recovery across studies, could impact the performance outcome, as the glycogen stores are typically restored to pre-exercise levels in 20–24 h, given that sufficient CHO is provided [1,2]. A subgroup analysis on TTE performance showed, when ingesting supplements during exercise and/or during recovery it significantly prolongs TTE performance. However, in the present meta-analysis, a significant effect on performance was only evident during long-term recovery periods (i.e., ≥8 h), no significant effect was found when the recovery period was short-term (i.e., ≤8 h). Three trials [10,15,17] assessed the effects of a long-term (i.e., ≥8 h) recovery between two exercise bouts. The result from these trials revealed a significant longer subsequent TTE (*p* = 0.001) after ingesting CHO-PRO compared to CHO (Figure 6). However, two of these trials [15,17] also provided supplementation during exercise. This could have had an additional effect in prolonging the performance duration to fatigue. In addition, when interpreting these results, one should also consider that only three trials examined the effect with long-term recovery. More studies implementing long-term recovery, when investigating the effect of CHO-PRO supplementation, are needed.

Furthermore, differences in a preceding exercise to deplete the muscle glycogen levels exists across trials. First, some studies (*n* = 8) [15,16,17,18,27,30,32,39] did not include a preceding exercise bout to deplete the muscle glycogen levels, which could impact the potential effect of adding protein to a CHO beverage. Second, in studies including a prior depletion protocol the exercise protocol varied across investigations (e.g., mode, intensity, and duration) (Table 1). In some studies, the preceding exercise bout was ≤70 min, which may not provide enough time to deplete the muscle glycogen content sufficiently. A ~50% decline in muscle glycogen content has been reported, when cycling at >70% VO2max for minimum 50 min [42], but very low levels of glycogen have mostly been shown, when cycling for >1.5 h [1,2,8].

In addition, differences in the exercise type employed to investigate athletic performance exists across the trials. Many studies have used TTE as the performance test. However, in some studies a TT performance has been employed. A TT performance test is considered a more relevant physiological test, as it is a highly reliable and repeatable test and an effective predictor of cycling performance [43,44]. To our knowledge this is one of the first meta-analysis to comprehensively assess the effect of CHO-PRO vs. CHO on both TTE and TT, respectively, including both long-term and short-term recovery periods. In the present review, we found a significant effect in TT performance, when ingesting CHO-PRO compared to CHO-only (Figure 10), with an improvement in performance by 3.39 min. The protocol design and duration of recovery varied across the pooled investigations using TT performance tests, these have likely contributed to the differences in performance outcome across the trials. The majority of studies conducted a preceding exercise bout [12,28,29,31,33,34,35,36,37,38], while some studies did not [27,30,32,39]. Moreover, differences in duration and intensity of the preceding exercise bout was evident, which could result in different muscle glycogen levels prior to and during the TT performance test between trials. In addition, the duration of recovery also varied. Some studies did not include a recovery period [12,27,30,32,33,37,38,39], whereas others implemented a short-term recovery [28,29,34,35,36] and in one trial the recovery time was long-term [31]. Furthermore, there were inter-study differences in the TT performance tests with regards to distance and modality (e.g., cycling, running). Variables such as gender and age of subjects could also affect TTE and TT performance outcome. Physiological and morphological characteristics may account for both gender differences (e.g., differences in muscle mass, body fat, aerobic capacity due to genetic and hormonal responses) and age-related differences seen in athletic performance. Only two of the investigations included in this review compared the performance differences in regard to gender. Interestingly, both found no significant differences in TTE performance between men and women [15,21]. In addition, muscle damage repair and endogenous glycogen restoration are possible mechanisms which have been linked to improved recovery and performance. Prior trials have shown that CHO-PRO administration can increase the rate of muscle glycogen synthesis beyond that of CHO alone [8,40] and hence may contribute to improved exercise performance in a subsequent bout. However, a study by Ferguson-Stegall [34] demonstrated an improved TT but no significant differences in glycogen re-synthesis when comparing CHO-PRO with CHO-only supplement. Furthermore, some investigators have proposed that reducing muscle damage that occurs during endurance exercise is a possible mechanism for improved performance with CHO–PRO ingestion. Saunders et al. [15,16] has reported significant improvements in TTE concomitant with significant reductions in markers of muscle damage when comparing CHO–PRO to CHO-only [15,16]. While others have reported significant reduction in muscle damage without an improvement in performance [17,18]. Hence, the association between muscle damage, glycogen restoration and improved cycling performance is not fully elucidated. A study by Ferguson-Stegall et al. [34] demonstrated an enhanced protein synthesis (i.e., upregulated mTOR activation) with CHO-PRO supplementation compared to CHO-only.

Other methodological differences across investigations include differences in the supplements provided. In some studies, beverages were matched for caloric content, while in others they were matched for CHO content. A subgroup analysis in this review demonstrated that providing a higher caloric content by adding protein to a carbohydrate-matched supplementation significantly improved performance time (Figure 9b), whereas an isocaloric content did not (Figure 8). In addition, when supplements were matched for volume only, no significant effect was found with CHO-PRO compared to CHO. Indicating, that a sufficient CHO content is important in improving performance.

Investigating the effect of the different types of protein and CHO on athletic performance was beyond the scope of the present review. However, the majority (*n* = 18 studies) of the investigations included in this review used whey as the protein source [10,11,12,13,16,17,18,20,21,22,24,26,30,31,32,35,36,37]. Proteins and specific amino acids (AA) have been shown to stimulate pancreatic secretion of insulin [45,46,47]. Especially, a synergistic effect has been observed by certain AAs to result in a potent stimulation of the β-cells [45,47]. Leucine, in particular, seems to be the potent stimulator of insulin secretion [8,45,48]. A study by Upshaw et al. [29] reported no effect in same-day-TT performance, when investigating the effect of different protein types, including animal- and plant-based proteins. In addition, a former study [49] observed a similar improved insulin sensitivity in type 2 diabetics, when comparing the effect of diets high in animal-based protein to diets high in plant-based protein. This suggests that the macronutrient and caloric content may be more important for athletic performance than the protein type. However, to our knowledge only a few studies have investigated the effect of plant-based protein on performance. Hence, more studies investigating the effect of different protein sources are therefore needed.

### 4.1. Future Research

The intent of this manuscript was to provide a systematic review of clinical trials investigating the effect of CHO-PRO on athletic performance compared to CHO. It is evident that methodological differences exist across trials to a great extent, which could explain the differences in performance outcome in the literature. Considering that the digestive properties and AA profile of proteins may differentially affect muscle protein synthesis and glycogen synthesis (by mediating insulin-secretion) [8,45,46,48], future research should assess whether manipulating the source of protein (e.g., animal- or plant-based) exerts enhanced effect on performance. Furthermore, more studies investigating long-term recovery, assessing muscle glycogen levels through biopsy techniques, as well as the timing of CHO-PRO intake (e.g., during exercise and/or recovery) are warranted.

Due to the need to consume beverages or gels containing high amount of sugars during sporting events, athletes have been considered as being at high risk for oral disease (e.g., carious lesions) [50,51]. Therefore, future research investigating the potential risk effects of energy supplements on dental health and risk of e.g., carious lesions in endurance athletes are needed, especially during sporting events lasting over several days, when the intake of high sugary supplements may be high.

### 4.2. Limitations

This review contains some limitations. First, only full-text articles written in English were included. Second, some of the trials tested the effect of treatment when subjects were fed while others when they were fasting, this could moderate the effect of CHO-PRO. Third, we did not take into account differences in the amount or type of protein provided between the trials. Fourth, when interpreting the results, one should consider the differences in the protocol design across studies. This includes the preceding exercise bout (e.g., duration and intensity) and timing of supplement intake, which could affect the level of glycogen depletion. Nevertheless, it was not possible to estimate the extent of glycogen depletion, based on the description in the included trials. In addition, not all studies measured the level of muscle glycogen via biopsies.

## 5. Conclusions

In the present Systematic Review and Meta-analysis, a total of 30 RCT studies were included, comprising 43 trials and 326 participants.

In conclusion, we found a significant overall effect in both TTE and TT performance, when CHO-PRO was ingested compared to CHO. A subgroup analysis demonstrated that this was significant when CHO-PRO was provided during and/or following an exercise bout. We found no significant effect in TTE, when the recovery time was short-term (i.e., ≤8 h), but TTE was significant, when a long-term recovery was implemented. Although the meta-analysis found no differences between CHO-PRO and CHO, individuals with a limited time to recover should ensure they have an adequate CHO intake to efficiently replenish glycogen deposits. Moreover, results demonstrate an ergogenic effect of CHO-PRO, offering performance benefit when protein is added to an optimal amount of CHO supplement (e.g., matched for CHO content) as opposed to supplements matched for caloric content.

## Figures and Tables

**Table 1:** Characteristics of included studies.

Citation, Year	Participants	Study Design	Preceding Exercise	Recovery Duration (hr)	Performance Exercise	Performance Duration (min)	Supplement Administration
Martinez-Lagunas et al. 2010	12 (5F, 7M) VO2max: 57.3 ± 2.7 Road cyclists/triathletes	Crossover DB	Cycling: 150 min, 55–75%VO2max	0	Cycling TTE 80% VO2max	(a) CHO-PRO: 30.5 ± 22.85 (b) CHO-PRO: 28.9 ± 25.17 CHO: 26.9 ± 23.63	255.4 ± 9.1 mL during EX every 20 min
Betts et al. 2007	6 M VO2max: 61.4 ± 7.3 Recreationally active	Crossover DB	Running: 70 min, 70% VO2max	4	Running TTE 70% VO2max	(a) CHO: 87.3 ± 16.9 (b) CHO: 99.9 ± 16.9 CHO-PRO: 91.2 ± 15.8	581 mL/hr during REC every 30 min
Saunders et al. 2004	15 M VO2peak: 52.6 ± 10.3 Cyclists	Crossover DB	(a) None (b) Cycling to fatigue; 75%VO2peak	0 12–15	(a) Cycling, TTE, 75%VO2peak (b) Cycling TTE; 85% VO2max	(a) CHO-PRO: 106.3 ± 45.2 CHO: 82.3 ± 32.6 (b) CHO-PRO: 43.6 ± 12.5 CHO: 31.2 ± 8.7	1.8 mL/kg every 15 min during EX, and 10 mL/kg PRE-EX
Romano-Ely et al. 2006	14 M VO2max: 59.8 ± 11.9 Physical active	Crossover DB	(a) None (b) Cycling to fatigue 70%VO2peak	0 22–24	(a) Cycling, TTE, 70% VO2peak (b) Cycling TTE; 80% VO2max	(a) CHO-PRO: 98.1 ± 28.7 CHO: 95.8 ± 29.7 (b) CHO-PRO: 42.9 ± 21.8 CHO: 42.3 ± 18.6	2 mL/kg every 15 min during EX, 10 mL/kg BW and 10 mL/kg PRE-EX
Saunders et al. 2007	13 (5F, 8M) VO2peak: 57.6 ± 6.7 Recreationally active	Crossover DB	None	0	Cycling TTE 70% VO2max	CHO-PRO: 116.6 ± 28.5 CHO: 102.8 ± 25	0.146g CHO/kg BW/serv and 0.0365g PRO/kgBW/serv during EX and PRE-EX
Williams et al. 2003	8 M VO2max: 62.4 ± 1.1 Trained cyclists	Crossover N.D.	Cycling: 120 min, 65–75%VO2max	4	Cycling TTE 85% VO2max	CHO-PRO: 31.1 ± 12.39 CHO: 20 ± 7.75	355 mL during REC at t = 0 and 2 hr PRE-EX
Ivy et al. 2003	9 M VO2max: 61.3 ± 2.4 Cyclists	Crossover DB	Cycling: 3 hr; 45–75% VO2max	0	Cycling TTE 85% Pmax	CHO-PRO: 26.9 ± 4.5 CHO: 19.7 ± 4.6	200 mL every 20 min during EX
Ferguson-Stegall et al. 2010	15 (7F, 8M) VO2max: 56.15 ± 1.02 Cyclists/triathletes	Crossover DB	Cycling: 180 min, 45–70% VO2max	0	Cycling TTE 74–85% VO2max	CHO-PRO: 31.06 ± 22.31 CHO: 26.03 ± 16.54	275 mL every 20 min during EX
Rustad et al. 2016	8 M VO2max: 69.6 ± 1.3 Endurance trained	Crossover DB	Cycling to fatigue, 72–90% VO2max	~18	Cycling TTE 72% VO2max	CHO-PRO: 63.5 ± 12.45 CHO: 49.8 ± 15.27	1.2 g/kg BW/hr during REC every 30 min during the first 2 hr of REC
Thomas et al. 2009	9 M VO2max: 59.1 ± 1.5 N.D.	Crossover DB	Cycling to fatigue, 60–90% Pmax	4	Cycling TTE 70% Pmax	(a) CHO-PRO: 32 ± 11 (b) CHO-PRO: 21 ± 8 CHO: 23 ± 8	1 g/kg BW during REC at t = 0 and 2 hr
Valentine et al. 2008	12 M VO2max: 53.4 ± 7.2 Active (incl. cycling)	Crossover DB	None	0	Cycling TTE 75% VO2max	CHO-PRO: 126.2 ± 25.4 (a) CHO: 117.5 ± 24.2 (b) CHO: 121.3 ± 36.8	250 mL every 15 min during EX
Richardson et al. 2012	11 (5F, 7M) M: VO2max: 50 ± 7.3 F: VO2max: 45.2 ± 3.9 Physically active (incl. cycling)	Crossover DB	Cycling TTE 75% VO2max	3	Cycling TTE 75% VO2max	CHO-PRO: 24 ± 13.44 CHO: 25.4 ± 11.77	1.5 g/kg BW/hr during REC every 30 min
Qin et al. 2017	10 M VO2max: 48.1 ± 8.4 Endurance runners/cyclists	Crossover DB	Cycling: 60 min, 70%VO2max	6	Cycling TTE 70% VO2max	CHO-PRO: 52.7 ± 15.03 (a) CHO: 55.3 ± 14.4 (b) CHO: 58.42 ± 11.1	1.8 mL/kg BW ever 15 min during EX, and 10 mL/kg PRE-EX
McCleave et al. 2011	14 F VO2max: 46.74 ± 1.6 Triathletes	Crossover DB	Cycling: 3 hr; 45–70% VO2max	0	Cycling TTE ~75% VO2max	CHO-PRO: 49.94 ± 26.23 CHO: 42.36 ± 23.24	275 mL every 20 min during EX
Karp et al. 2006	9 M VO2max: 65 ± 9 Cyclists	Crossover SB	Interval cycling workout	4	Cycling TTE 70% VO2max	(a) CHO-PRO: 40 ± 14.7 (b) CHO-PRO: 26.3 ± 10.3 CHO: 41.3 ± 15	509.1±36 mL during REC
Alghannam et al. 2016	6 (5M, 1F) VO2max: 64 ± 4 Recreational runners	Crossover DB	Cycling TTE 70% VO2max	4	Cycling TTE 70% VO2max	CHO-PRO: 49 ± 15 CHO: 51 ± 13	10 mL/kg/hr during REC
Osterberg et al. 2008	13 M VO2max: 56 ± 0.12 Cyclists	Crossover DB	Cycling ~120 min SS	0	Cycling TT 7kJ/kg BW	CHO-PRO: 38.8 ± 5.5 CHO: 37.1 ± 3.8	250 mL every 15 min during PRE-EX
Saunders et al. 2009	13 M VO2max: 60.8 ± 1.6 Recreational cyclists	Crossover DB	None	0	Cycling TT 60 km	CHO-PRO: 134.3 ± 16.2 CHO: 135.1 ± 14.8	200 mL every 5 km and 500 mL PRE-EX
Hall et al. 2013	10 M VO2max: 66.2 ± 6 Cyclists	Crossover DB	Interval cycling workout ~2.5 hr	4	Cycling TT 7kJ/kg BW	CHO-PRO: 30.2 ± 4.93 CHO: 30.82 ± 5.73	250 mL every 15 min during PRE-EX, 30 mL every 5 min during TT, and recovery supplement
Upshaw et al. 2016	8 M VO2max: 61.2 ± 1.4 Cyclists	Crossover DB	Cycling intervals	4	Cycling TT 20 km	CHO: 37.85 ± 2.1 (a) CHO-PRO: 34.58 ± 2.5 (b) CHO-PRO: 34.88 ± 1.1 (c) CHO-PRO: 34.47 ± 1.7 (d) CHO-PRO: 34.83 ± 2.2	CHO 247 kJ and CHO-PRO 2.107 kJ during REC
Gui et al. 2017	11 F VO2max: 49 ± 6.6 Recreational runners	Crossover DB	None	0	Running, TT 21 km	CHO-PRO: 132.4 ± 11.5 CHO: 129.6 ± 8.8	150 mL every 2.5 km during EX
Sollie et al. 2018	8 M VO2max: 74 ± 1.6 Cyclists	Crossover SB	Cycling to fatigue, interval + sprint, 50–90% VO2max	~18	Cycling; Preloaded TT 30 min at 73% W and TT	CHO-PRO: 41.53 ± 1.51 CHO: 45.26 ± 1.32	7.06 mL/kg BW/hr during the first 2 hr of REC
Finger et al. 2018	13 M VO2max: 62.2 ± 5.4 Amateur athletes	Crossover DB	SDT: 10 km running 40 km cycling	0	Duathlon, TT 5 km running	CHO-PRO: 21.12 ± 2.315 CHO: 21.17 ± 2.175	150 mL at 5, 20 and 35 km during 40 km cycling (PRE-EX)
van Essen et al. 2006	10 M VO2max: 63 ± 2 Cyclists/triathletes	Crossover DB	None	0	Cycling TT 80 km	CHO-PRO: 135 ± 6.3 CHO: 135 ± 6.3	250 mL every 15 min during EX
Breen et al. 2010	12 M VO2max: 62.7 ± 6.3 Cyclists	Crossover DB	Cycling; 120 min, 55% VO2max	0	Cycling TT (880±27 kJ)	CHO-PRO: 60.51 ± 8.3 CHO: 60.13 ± 4.5	270 mL 15 min of PRE-EX
Ferguson-Stegall et al. 2011	10 (5F, 5M) F: VO2max: 47.6 ± 1.5 M: VO2max: 57.7 ± 2.8 Cyclists	Crossover DB	Cycling; 1.5 hr at 70% VO2max + 10 min intervals	4	Cycling TT 40 km	CHO-PRO: 79.43 ± 2.11 CHO: 85.74 ± 3.44	1000–1400 mL during REC
Saunders et al. 2018	16 (11F, 5M) N.D. Amateur athletes	Parallel study DB	None	0	Marathon run, TT	CHO-PRO: 236.8 ± 26.4 CHO: 244.6 ± 35.7	4.5 ± 1.4 gels CHO and 5.9±1.5 gels CHO-PRO during EX
Goh et al. 2012	12 M VO2max:65 ± 7 Cyclists	Crossover DB	Interval cycling workout ~1 hr	4	Cycling: 20 min at 70% VO2max + 20 km TT	(a) CHO-PRO: 48.8 ± 7.2 (b) CHO-PRO: 50.3 ± 9.3 CHO: 48.5 ± 5.1	750 mL during REC
Wolfe et al. 2016	10 (2F, 8M) VO2max: 54.6 ± 6.5 Cyclists/Triathletes	Crossover DB	Cycling, TT 40 km	0.5	Cycling, TT 10 km	CHO-PRO: 23.9 ± 1.0 CHO: 24 ± 0.9	1925 mL during the protocol
Oosthuyse et al. 2015	8 M VO2max: 60.9 ± 5.1 Cyclists	Crossover DB	Cycling; 2 hr at 60% Wattmax	0	Cycling, TT 16 km	(a) CHO-PRO: 27.37 ± 2.29 (b) CHO-PRO: 27.53 ± 2.01 CHO: 28.12 ± 2.53	400 mL prior EX and 200 mL every 15 min during EX

DB: double-blind; SB: single-blind; SS: steady-state; M: male; F: female; N.D.: not described; CHO: carbohydrate; CHO-PRO: carbohydrate and protein; TT: time-trial; TTE: time-to-exhaustion; EX: exercise, PRE-EX: preceding exercise; REC: recovery; Incl.: including; BW: Bodyweight (kg); t: timepoint; Min: minutes; hr: hour; VO2max: mL/min/kg.

## Supplementary Materials

The following are available online at https://www.mdpi.com/2072-6643/12/5/1483/s1, Appendix S1. Search-terms in PubMed and Embase, Figure S1 and S2. Funnel plots of publication bias. S1: TTE and S2: TT performance.
