# Supplementary material for: The Effect of Ingesting Carbohydrate and Proteins on Athletic Performance: A Systematic Review and Meta-Analysis of Randomized Controlled Trials
Source: Nutrients. 2020 May 20;12(5):1483. doi: 10.3390/nu12051483 (PMC7284704; doi:10.3390/nu12051483)
Supplement: Supplementary file 1 [file nutrients-12-01483-s001.zip › Supplementary files/Supplementary Appendix S1 Search Terms EMBASE and PubMed.pdf]

Appendix 1 – Search terms

Full search terms used in PubMed and EMBASE. Articles within the following publication dates: 01.01.1950-31.10.2019, were included.

PubMed:

("carbohydrate" OR "glucose" OR "fructose" OR "sucrose" OR "protein" OR "amino acid" OR "glycogen" OR "Carbohydrate-protein" OR "carbohydrate protein complex" OR "carbohydrate-protein supplement") AND ("beverage" OR "drink" OR "dietary proteins") AND ("performance" OR "cycling" OR "cycle" OR "bicycling" OR "time to exhaustion" OR "running" OR "time trial" OR "recovery" OR "postexercise" OR "endurance" OR "muscle" OR "physical endurance" OR "cycling time" OR "time factors" OR "Running/physiology" OR "Restitution*") AND (athlete* OR kinetics OR healthy)* ***Sort by: Author Filters:*** *Randomized Controlled Trial; Clinical Trial*

EMBASE:

('human'/exp OR 'normal human'/exp OR 'adult'/exp) AND ('carbohydrate diet'/exp OR 'carbohydrate diet' OR 'diet, carbohydrate' OR 'diet, high carbohydrate' OR 'dietary carbohydrate' OR 'dietary carbohydrates' OR 'high carbohydrate diet' OR 'carbohydrate intake'/exp OR 'alimentary carbohydrate' OR 'carbohydrate intake' OR 'carbohydrate, alimentary' OR 'carbohydrate, dietary' OR 'diet carbohydrate' OR 'saccharide intake' OR 'glucose/exp' OR 'protein diet'/exp OR 'protein intake'/exp OR 'diet protein' OR 'diet, protein' OR 'dietary protein' OR 'dietary proteins' OR 'egg proteins, dietary' OR 'fish proteins, dietary' OR 'food protein' OR 'intake, protein' OR 'plant proteins, dietary' OR 'protein consumption' OR 'protein feeding' OR 'protein food' OR 'protein intake' OR 'protein nutrition' OR 'sports drink'/exp) AND ('treadmill exercise'/exp OR 'running'/exp OR 'running' OR 'exercise'/exp OR 'cycling'/exp OR 'bicycling' OR 'cycling' OR 'physical performance'/exp OR 'beverage') AND ('time'/exp OR 'convalescence' OR 'exhaustion'/exp OR 'exhaustion' OR 'fatigue'/exp OR 'exercise test'/exp OR 'exercise tolerance'/exp OR 'endurance'/exp OR 'glycogen muscle level') AND ('randomized controlled trial'/exp OR 'clinical study' OR 'human experiment' OR 'exercise recovery'/exp OR 'double blind procedure'/exp OR 'randomization') AND 'article'/it
