# Supplementary figures and images for: The Effect of Ingesting Carbohydrate and Proteins on Athletic Performance: A Systematic Review and Meta-Analysis of Randomized Controlled Trials
Source: Nutrients. 2020 May 20;12(5):1483. doi: 10.3390/nu12051483 (PMC7284704; doi:10.3390/nu12051483)

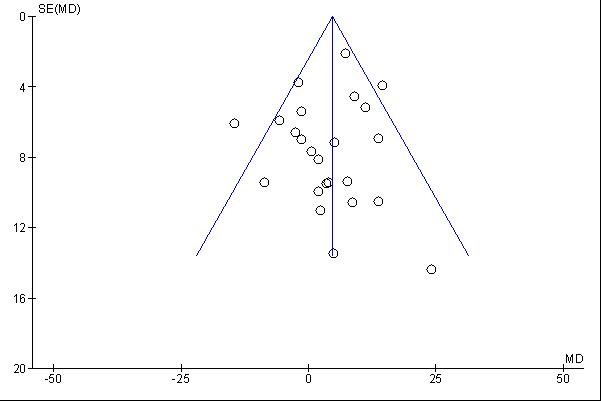

Supplement: Supplementary file 1 [file nutrients-12-01483-s001.zip › Supplementary files/Supplemental Figure S1 TTE.jpg]

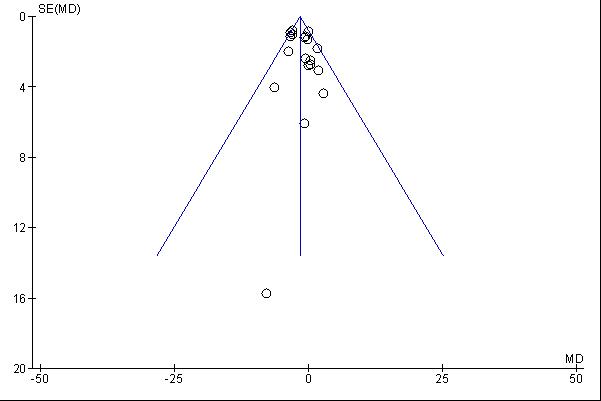

Supplement: Supplementary file 1 [file nutrients-12-01483-s001.zip › Supplementary files/Supplemental Figure S2 TT.jpg]
